# The Relation of the Physician to the Purity of the Articles Used in Prescriptions

**Published:** 1867-10

**Authors:** 


### CHICAGO MEDICAL JOURNAL.

VOL. XXIV. OCTOBER, 1867. No. 10.

### Original Contributions.

### THE RELATION OF THE PHYSICIAN TO THE PURITY OF THE ARTICLES USED IN PRESCRIPTIONS.

There is in existence a work, entitled the "United States Dispensatory," which is assumed to be the standard upon which physicians base their doses of remedies, and from which the dispenser derives his formulæ for the manufacture of the articles prescribed. It contains the elements of chemical manufacture and materia medica, and is the *only guide* which the pharmaceutist of the United States should recognize.

Since the war has brought its heavy taxations, the tendency to adulterate everything, not only in food but also in medicine, has more and more increased. The higher the price, the greater the temptation.

For several years past, we have met in commerce with spirits of nitre of a perfectly limpid, colorless appearance, totally at variance with the description of the pharmacopœia. For instance, this authority says: "it should be a pale yellow volatile liquid, of fragrant, etherial odor, and pungent, aromatic taste. Its officinal gravity is .837, and when heated, by means of a water bath, the sweet spirits of nitre begins to boil at 145°."

Dr. Edward R. Squibb, of New York, has examined samples of commercial spirits of nitre, and found them to contain the nitric ether in the following proportion: four, between 1 and 2 per cent, and one under 1 per cent, while the standard laid down by the U.S.P. says, there should be present at least $4\frac{3}{10}$

per cent. Here we have a sad display of the deficiency of strength in some of these *handsome, clear preparations*, which are sold wholesale over the country.

But the absence of the full proportion of ether is not their only means of adulteration, they can so handsomely balance, with water and alcohol, the specific gravities, that it *shall preserve the specific gravity of the Pharmacopœia* and, perhaps, not have *one-fourth of its remedial agent—the hyponitrous ether—present.* So far, we physicians have to contend, in reference to this one article.

Again, take another article—chloroform. No one article in the Pharmacopœia should be more jealously guarded against adulteration, and yet, on account of the immense amount of material consumed to procure a small amount of chloroform—100 ℔s. of chloride of lime producing but 5 to 6 ℔s. of chloroform; it is subject, also, to adulteration. So very impure is the chloroform that we usually procure from commerce, (excepting that *purified* by Dr. Squibb and Dr. Duffield,) that the United States Dispensatory has placed on the primary list the *chloroformum purificatum.* Dr. Squibb, of New York, has already called the attention of the profession to the danger of using *impure* chloroform, and a pure article made by Dr. Duffield, of Detroit, and tried and used by Prof. Gunn, went begging for purchasers for three years, because it could not be afforded at the price of the impure article.

It will not do for the physician to calculate upon ordinary commercial strengths, as occasionally he may find his prescriptions will be put up with the genuine article and trouble, therefore, will result, as his dose was based not on a strong article, but upon the commercial article. The druggist has become, in our country, a strictly commercial man, discarding, in most cases, science, and operating with drugs solely with an eye to pecuniary profit. There are very few among our Western pharmacists who have ever had a chemical education and who are, therefore, competent to test articles, as regards their purity. How is such a person able to tell whether his Dover's powders are made from opium containing $12\frac{40}{100}$ per cent of morphia, or from leached opium, containing less than 1 per cent of the alkaloid. Our American law does not recognize a "State Board of Examination," such as exists in Prussia and Bavaria, and before which the apothecary must pass examination before he is recognized as fit for dispensing medicines.

Our sole remedy or control in this matter lies with the physician. It is useless to try and educate a pharmacist *after* he is immersed in business and laden with business cares. He cannot take time then to study up chemical principles and means of detection, and attend to the sales of his store too.

The physician, on the other hand, has a perfect control of this matter; he sees very quickly whether the fluid extract verat. viride is doing service in a case of pneumonia or of acute bronchitis, by the reduction of the pulse; he knows whether he is getting the effect of the conium or hyoscyamus, by the state of the patient, dilatation of the pupil, etc. It is a mistaken idea that some physicians have of generosity, in excusing ignorance on the part of druggists, as if this very ignorance detracted from the moral culpability of selling adulterated drugs. It is looked upon as terrible if a patient dies from an *overdose* of medicine—he is poisoned; but should the medicine be not fully up to the requirement of the Pharmacopœia, and the physician, losing time in getting the effects of the medicine, finds the disease has passed from the *critical* state into the *fatal* termination, should not the remedy and its dispenser be held accountable for the death which could have been avoided had the medicine been full strength?

Let the profession wake up to this fact and test in their practice the preparations of the market, take men who are reliable in conscience as well as in science, and compel these apothecaries to reject every inert preparation, substituting those which show their therapeutic power in their hands.

It would not be long before this influence, emanating from the Faculty, would pass beyond the retailer, to the wholesaler, and better classes of goods, drugs of the first quality, could be found in every store, and empiricism and kindred delusions be driven from our midst, and the science of medicine, upheld by conscientious efforts in her collateral departments, will advance steadily to the goal she is now striving to reach—the amelioration and exaltation of the human race.—[Com.

